# The pathogenicity of *Staphylococcus epidermidis* on the intestinal organs of rats and mice: an experimental investigation

**DOI:** 10.1186/1471-230X-14-126

**Published:** 2014-07-12

**Authors:** Ezekiel Olugbenga Akinkunmi, Oluwole Isaac Adeyemi, Oluwatoyin Abimbola Igbeneghu, Esther Omowunmi Olaniyan, Abidemi Emmanuel Omonisi, Adebayo Lamikanra

**Affiliations:** 1 Department of Pharmaceutics, Faculty of Pharmacy, Obafemi Awolowo University, Ile-Ife, Nigeria; 2 Department of Pharmacology, Faculty of Pharmacy, Obafemi Awolowo University, Ile-Ife, Nigeria; 3 Department of Morbid Anatomy and Forensic Medicine, Obafemi Awolowo University Teaching Hospital Complex, Ile-Ife, Nigeria

**Keywords:** Pathogenicity, Enteric infection, Staphylococci, Gastrointestinal tract

## Abstract

**Background:**

*Staphylococcus epidermidis* is the most frequently isolated species of the coagulase negative staphylococci from human stool. However, it is not clear how its presence in the gut affects the cellular structures and functions of this organ. In this study therefore, the pathogenicity of strains of *S. epidermidis* which were isolated from the stool samples of apparently healthy children was investigated in mice and rats.

**Methods:**

The albino mice (22—30 g) and albino rats (100-155 g) of both sexes were infected orally and intraperitoneally with graded doses of the bacteria and subjected to behavioral and histopathological examinations.

**Results:**

Acute infection in these animals caused temporary behavioural changes as shown by restlessness and abdominal stretchings but did not result in death even at a dosage of 2 × 10^9^ cfu/kg. Daily administration of the same dose for 14 days resulted in the death of 11 out of 21 (52.4%) mice. Histopathological examination of the affected organs showed congestions, aggregations and multinucleated hepatocytes in the liver, infiltration of the kidney tubule interstitial by chronic inflammatory cells, coagulative necrosis of the kidney, spleen, intestine and stomach cells as well as marked stroma fibrosis of the spleen. Coagulative necrosis of cells was the most frequently occurring pathological alteration. Lethality and pathological effects reflected the virulence factors expressed by the organism which are biofilm formation, haemagglutination properties and capsule production.

**Conclusions:**

The results indicate that strains of *S. epidermidis* colonising the gut can cause serious pathological changes on certain organs such as kidney, liver, intestine, stomach and spleen which, depending on their severity, could be fatal.

## Background

Coagulase-negative staphylococci (CoNS) are a prominent part of the normal flora of the human skin. They have been isolated from different body sites including the mucous membranes such as the nose, throat, vaginal wall and the gastrointestinal tract [1-3]. CoNS colonising several niche in the human body are recognised as established pathogens playing great roles in different human infections [4,5]. However, the pathogenic roles of CoNS colonising the gastrointestinal tract is not very well established.

*S. epidermidis* is a well-characterised, nonfastidious CoNS most commonly isolated in the clinical microbiology laboratory [6]. It is one of the most important nosocomial pathogens associated with catheter-related and other indwelling medical device-related infections [5,6]. The production of slime and the ability to form biofilm has therefore been reported to play the most important roles in the pathogenesis of *S. epidermidis* and other CoNS-induced infections [5].

Various workers have indicated that *S. epidermidis* is associated with infections to various degrees [6,7]. It has been reported that *S. epidermidis* was among the CoNS accounting for 86% of clinically significant infections caused by CoNS recovered from non-sterile sites [7]. An earlier report have established a 100% endocarditis incidence in rats injected with *S. epidermidis*[8]. Further studies have indicated that clinical strains of *S. epidermidis* were pathogenic, virulent and invasive after intraperitoneal infection in mice causing macroscopic pathological changes in the spleen, kidney, liver, and the peritoneum [9]. However, reports from another study could not demonstrate pathogenicity in *S. epidermidis* in a mouse mastitis model even when a large dose of inoculum was used [10]. An earlier report had indicated that intraspecies differences in virulence do occur in this organism [11].

*S. epidermidis* is a prominent CoNS isolated from the gastrointestinal tract of children [2,4]. Isolates from the intestinal tracts have been found to express arrays of virulence factors that promote staphylococcal virulence indicating their pathogenicity potentials [12,13]. These virulence determinants include toxins and enzymes such as those which have been associated with *S. aureus*[13].

This study investigated the pathogenicity of strains of *S. epidermidis* originally isolated from the faeces of apparently healthy children on the intestinal organs of experimentally infected mice and rats.

## Methods

### Bacterial isolates

The *S. epidermidis* isolates used in this study were recovered from the stool samples of apparently healthy children in Nigeria as reported previously [13]. Three strains of the organism were used. The first isolate (A245A) was from a child attending a Day-Care in the community and in which the isolate was shown to express three virulence factors comprising of haemolysin production, encapsulation, and biofilm formation. The second (A80A), showing two virulence factors comprising haemagglutination and biofilm formation, was from a child who was brought for immunization in a commuity health centre while the third (A246A), showing one virulence factor expressed as biofilm production, was from a child from the same health centre [13] (Table 1). Parental consent was obtained for each of the children used in the study.

**Table 1:** **Properties of the***S. epidermidis***strains used in this study**[13]

**Organism code**	**Properties**
A246A	Biofilm formation
A80A	Haemagglutination + Biofilm formation
A245A	Haemolysin production + Biofilm formation + Encapsulation

### Test animals

The study was carried out following approval from the Health Research and Ethics Committee, Obafemi Awolowo University, Ile-Ife, Nigeria. Albino mice (22—30 g) and albino rats (100-155 g) of both sexes were used for the study. They were reared in the animal house of the Faculty of Pharmacy, Obafemi Awolowo University Ile-Ife. The animals were kept in cages in the animal house with access to food and water *ad libitum* but were transferred to the experimental laboratory 8 hours before the experiment on each experimental day.

### Preparation of bacterial suspension for use in the infection of the rodents

The organisms were grown overnight in nutrient broth (Oxoid, England) contained in test tubes. The cultures were then adjusted to give the inoculum size ranging from 1 × 10^8^ cfu/ml to 1 × 10^9^ cfu/ml before administration into the animals through the oral or the peritoneal route as may be required.

### Acute animal infections

Effect of acute infection was determined in rats and mice using the acute toxicity testing method as proposed [14]. The experiments involved three different stages.

Nine rats and nine mice each were used for the first stage of the experiment. They consist of three animals per group, one group for each of the three strains of *S. epidermidis*. Each of the animals was weighed before the commencement of the experiment as the number of organisms administered to each of them was calculated relative to weight. For example, 0.5 ml of the inoculum of the bacteria isolates, containing approximately 1 × 10^8^ cells/ml was administered to rats weighing between 100-125 g while 0.6 ml was administered to those weighing between 140-155 g orally using pyrogen-free sterile oral catether to give a dose of approximately 5 × 10^8^ cfu/kg body weight of animal. All animals were observed closely for the first 6 hours, for every hour in the remaining part of the first day and then daily for 3 days. The times and types of any behavioural changes and adverse effects were noted.

For the second stage of the experiment three animals were used each for rats and mice (one animal per group) [14]. Each animal was given a dose of organisms that was twice that which was used in the first stage as described above. The animals were observed as in the first stage of the experiment.

Similarly, for the third stage, three animals were used. They were grouped into three sets. This time the isolates (1 × 10^9^ cfu/kg) were administered intraperitoneally using pyrogen-free sterile disposable syringes and the animals were observed as described above.

### Subacute toxicity tests

Four (4) groups of seven animals each were used for the experiment. One group served as the control in which only distilled water were given while the other three (3) groups were administered orally with the innoculum of the three organisms (5 × 10^8^ cfu/kg body weight) daily for 14 days. Adequate animal feed and water were given *ad libitum*.

For the experimental groups, the animals were observed closely within the first 24 hours after the administration of the culture. Doses were repeated each day for the fourteen day period of the experiment.

### Organ harvest

Organ harvest of the gastrointestinal tract was done each day for all the animals that died during the experimental period. Animals which survived the 14 days infection as well as those in the control group were sacrificed on the 15^th^ day by euthenizing under diethylether anaesthesia. The liver, kidneys, stomach, intestines and spleen were harvested and preserved in 10% buffered formalin for histopathological examination.

### Histopathological study

For the histopathological analysis, the specimens were fixed in 10% buffered formalin, dehydrated and passed through increasing concentrations of alcohol. Thereafter clearing was done by removing alcohol with use of xylene. The specimens were later embedded in wax and cut with rotary microtome to a thickness of 3 to 5 micrometer. They were allowed to float in a water bath to make them grease free and then mounted on a glass slide. The wax was removed with xylene, the samples were then rehydrated with alcohol in decreasing concentrations. Staining was done using haematoxylin and eosin (H & E staining). The specimens were examined under the microscope (Leica Microsystems Digital Imaging, Germany) and images were captured on a Leica DFC 280 CCD camera.

## Results

### Acute infection in mice

Generally, the behavioral responses of both mice and rats were similar after acute infection by the CoNS specie. In the first stage of the experiment, all the mice were active for the first few minutes after oral infection with the bacterial isolates. All the mice displayed intense grooming, reduced locomotion, lethargy and were sedated when compared with the control. None of the animals died throughout the three days of the experiment.

In the second stage of the work, after oral administration, the animals showed signs of discomfort, had reduction in activity and slept intermittently. The mouse administered with strains A245A convulsed and died 3 hours five minutes after the administration of the inoculum. Death was preceded by apnoea and diarrhoea which lasted over a period of 90 minutes.

### Acute infection in rats

For the rats, particularly within the first thirty minutes, all the test animals showed increased grooming, locomotor activity, rearing, and abdominal stretching when compared with the control. This was followed by about fifteen minutes display of reduced grooming, locomotor activity, rearing and sedation, which was more prominent in those administered with the bacteria strain A80A, before the animals regained normal activity.

All the animals huddled together within the first 24 hours. On the second day, they could not eat and were all lying on their stomach. By the third day however, their activity had been restored. All the rats also survived the second stage of the experiment, although they initially showed signs of discomfort and sedation.

### Results of sub-chronic infection

Within the first 24 hours of the experiment, all the mice in the test groups were observed to have reduced motor activities for about 15 to 30 minutes coupled with reduced feeding. After this, normal activities such as grooming, rearing, locomotion and feeding resumed.

The pattern of death of the mice on sub-chronic ingestion is shown in Tables 2 and 3. The total number of death in mice fed with strains A246A, A80A and A245A were 3, 4 and 4 respectively.

**Table 2:** Acute toxicity of strains of CoNS in rodents

**Organism Code**	**Death pattern at 3 days after oral administration in mice**		**Death pattern at 3 days after oral administration in rats**		**Death pattern at 3 days after intraperitoneal administration in rats**
	**1**^**st **^**stage**	**2**^**nd **^**stage**	**1**^**st **^**stage**	**2**^**nd **^**stage**	**3**^**rd **^**stage**
A246A	0/3	0/1	0/3	0/1	0/1
A80A	0/3	0/1	0/3	0/1	0/1
A245A	0/3	1/1	0/3	0/1	0/1

**Table 3:** **Mortality of mice after sub**-**chronic oral ingestion** (**Test group**, **n** = **7**)

**Organism Code**	**No of deaths on each day of 14 days of ingestion**														
	**1st**	**2nd**	**3rd**	**4th**	**5th**	**6th**	**7th**	**8th**	**9th**	**10**^**th**^	**11**^**th**^	**12th**	**13th**	**14th**	**Total No of death**
A246A	0	1	0	0	1	0	0	0	0	0	0	1	0	0	3
A80A	0	2	0	0	0	0	0	1	0	1	0	0	0	0	4
A245A	0	1	0	1	0	0	0	0	1	1	0	0	0	0	4

### Results of histopathology

**Figure 1:**
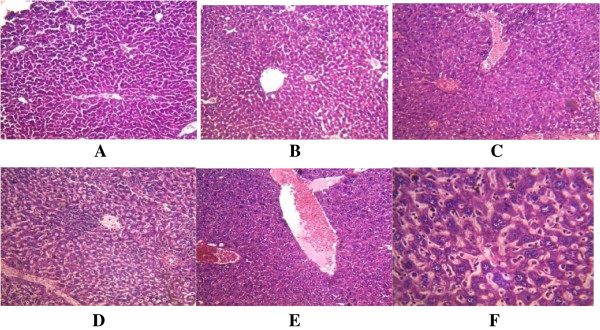
**Histological appearance of the liver. A**. The control, showing no histopathological changes. **B**. Mice infected with A246A* showing infiltration by parenchyma cells. **C**. Mice infected with A80A* showing marked mild infiltration of portal tract by chronic inflammatory cells and congestion of blood vessel. **D**, **E**, **F**. Mice infected with A245A* showing: **D**. aggregates. **E**. congestion. **F**. multinucleated hepatocytes. *As shown in Table 1.

**Figure 2:**
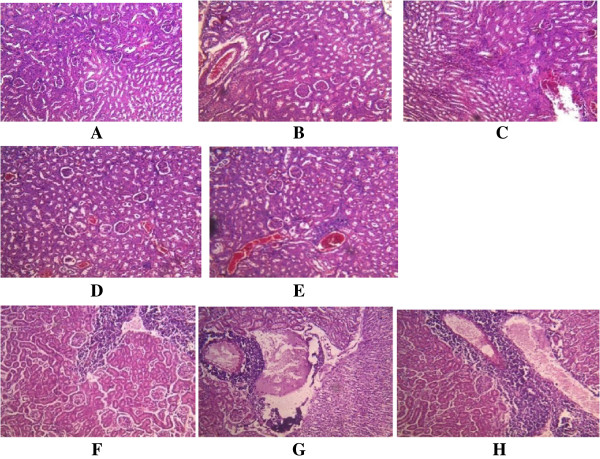
**Histological appearance of the kidney. A**. The control, showing no pathological changes. **BC**. Mice infected with A246A* showing **B**. essentially unremarkable, normal cells **C**. minimal infiltration of interstitial by inflammatory cells. **DE**. Mice infected with A80A* showing **D**. inflammation from mild to moderate **E**. infiltration of tubule interstitial, also congestion of vessel and mild compression of capillary luminal. **FGH**. Mice infected with A245A* showing **F**. kidney glomerulli with essentially unremarkable cells. **G**. necrosis **H**. intense infiltration on the tubule interstitial by chronic inflammatory cells. *As shown in Table 1.

**Figure 3:**
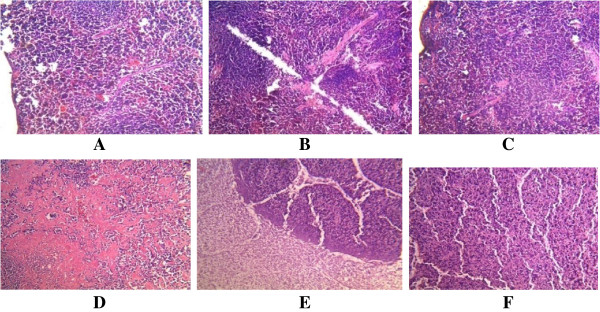
**Histological appearance of the spleen. A**. The control, showing no pathological changes. **BC**. Mice infected with A246A* showing mild destruction of the normal architecture of the spleen. **D**. Mice infected with A80A* with marked stroma fibrosis with dilatation of cyanocil. **EF**. Mice infected with A245A* showing **E**. area of coagulative necrosis **F**. marked destruction of the architecture of the spleen. *As shown in Table 1.

**Figure 4:**
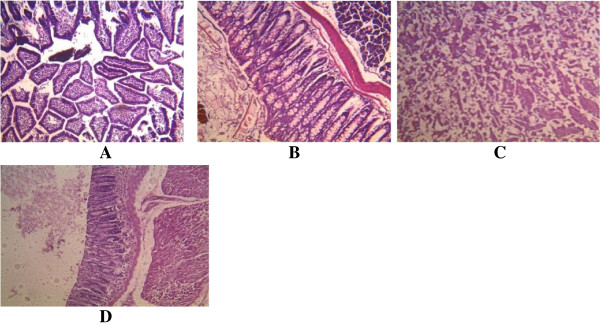
**Histological appearance of the intestine. A**. The control, showing no pathological changes. **B**. Mice infected with A246A* showing mild infiltration by inflammatory cells. **C**. Mice infected with A80A* showing coagulative necrosis of the gland. **D**. Mice infected with A245A* showing mild coagulative necrosis with reactive glandula dysplacia. *As shown in Table 1.

**Figure 5:**
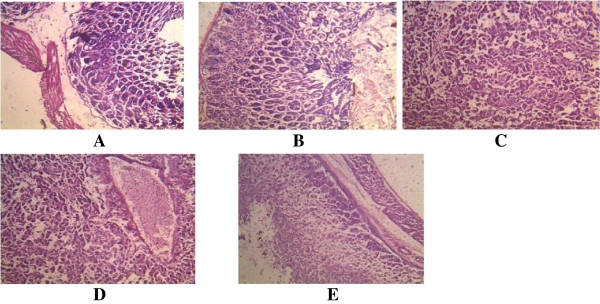
**s appearance of the stomach. A**. The control, showing no pathological changes. **B**. Mice infected with A246A* showing mild coagulative necrosis. **CD**. Mice infected with A80A* showing **C**. tubular necrosis **D**. acute necrotizing inflammation (coagulative necrosis). **E**. Mice infected with A245A* showing coagulative necrosis. *As shown in Table 1.

All the three strains of *S. epidermidis* tested in mice were found to exhibit striking histopathological changes to the gastrointestinal organs (Table 4) (Figures 1, 2, 3, 4 and 5). In the case of the liver, the cells showed congestion, aggregates and multinucleated hepatocytes. There were infiltration by parenchyma cells and mild infiltration of portal tract by chronic inflammatory cells. There were also signs of congestion of blood vessels (Figure 1). The kidneys exhibited from mild to moderate infiltration of the tubule interstitial cells by chronic inflammatory cells. In some cases there were necroses in addition to the mild compression of the capillary luminal. Some cells in the glomerulli appeared essentially normal and were not affected by the infections of the organisms notwithstanding the type and number of virulence factors (Figure 2). Coagulative necrosis was observed to be the most common pathological changes in the spleens, intestines and stomachs (Figures 3, 4 and 5). The spleen, in addition, showed mild and marked destruction of the normal architecture with marked stroma fibrosis and dilation of the cyanocil in certain cases. No pathological lesions were observed in any of the control animals.

**Table 4:** **Histopathological effects after sub**-**chronic oral ingestion** (**Test group**, **n** = **7**)

**Organism Code**	**Histopathological effects (Number of affected animals/Degree of damage)**				
	**Liver**	**Kidney**	**Spleen**	**Intestine**	**Stomach**
Control	No observable histopathological changes	No observable histopathological changes	No observable histopathological changes	No observable histopathological changes	No observable histopathological changes
A246A	Essentially unremarkable normal cells (2/-) Infiltration by parenchyma cells (5/+)	Essentially unremarkable normal cells (2/-) Infiltration of tubule interstitial (5/+)	Destruction of the normal architecture (7/+)	Essentially unremarkable normal cells (1/-) Infiltration by inflammatory cells (6/+)	Essentially unremarkable normal cells (2/-) Coagulative necrosis (5/+)
A80A	Infiltration of portal tract by chronic inflammatory cells and congestion of blood vessel (7/+)	Inflammation (3/+) Infiltration of tubule interstitial (4/++)	Stroma fibrosis with dilatation of cyanocil (7/+++)	Essentially unremarkable normal cells (1/-) Coagulative necrosis of the gland (6/++)	Tubular necrosis (3/++) Acute necrotizing inflammation (4/+)
A245A	Aggregation (1/+) Congestion (3/++) Multinucleated hepatocytes (3/++)	Essentially unremarkable normal cells (1/+) Necrosis (4/++) Infiltration of tubule interstitial (2/++)	Coagulative necrosis (2/++) Destruction of the architecture (5/+++)	Coagulative necrosis (3/+) Coagulative necrosis with reactive glandula dysplacia (4/++)	Tubular necrosis (1/++) Coagulative necrosis (6/++)

- = no observable effect, + = observable effect, ++ = clear histopatholigical damage, +++ = severe damage.

## Discussion

Oral administration of these organisms were undertaken to simulate what would obtain as a result of gastrointestinal colonization by these organisms. *S. epidermidis* isolates from faecal samples of young children demonstrated invasiveness to the gastrointestinal tract in mice and rats with intact immune system. From this observation, this organism may be an important epidemiological agent associated with the dysfunction of the tissues and organs of the gut.

Rodent models, particularly mice, are generally accepted and used as correlates of physiological and behavioral dispositions of humans. One of the obvious advantages of using mice is that the human and mouse genomes have been found to be very similar, so that many human genes have counterparts in mice [15,16]. Many organs in mice have also been found to be very similar to those in humans [15,16]. Thus, mice and rats have been used most frequently to study experimental coagulase negative staphylococcal infections as rough correlates of human infections [9,11], hence their use in this study. Acute oral administration of these organisms to the rodents could not produce immediate lethality in most cases but produced symptoms such as reduced motor activities, loss of appetite, intense grooming and abdominal discomfort. These anxiety-related responses might also suggest effects on the excitatory centres of the central nervous system [17].

As early as 1957, the minimum pathogenic dose for virulent staphylococci had been estimated to be of the order of 2 to 8 × 10^6^ organisms [18]. Similar bacteria doses ranging from 2 × 10^8^ cfu to 2 × 10^9^ cfu/kg body weight of isolates of *S. aureus* administered intraperitoneally were used earlier to produce 100% mortality in mice with death occurring within 5–10 hours [19]. This dose range could not produce such level of mortality in this study possibly confirming the well known report that *S. epidermidis* is less pathogenic than *S. aureus*[13].

On the other hand, the resulting mortality observed in a mouse after oral ingestion of one strain of *S. epidermidis* suggests the ability of these organisms to cause death after acute infection. In addition to this, the discovery that these dose ranges resulted in the serious histopathological lesions in these animals is a confirmation of the virulence of this organism in certain conditions and it also suggests the usefulness of such doses in determining its pathogenic potential.

The three strains of *S. epidermidis* tested in mice induced striking histopathological changes in the gastrointestinal organs. These pathological lesions, although varying in severity, were qualitatively consistent. The invasive nature of the histopathological lesions suggests the involvement of toxins. In addition to this, the pathological changes were similar to the known classic histopathological changes that are associated with *S. aureus*[20,21].

These observations help in confirming the roles virulence factors like haemagglutination, biofilm, capsule and haemolysin expressed by strains of this organism play in pathogenicity [13]. Haemaglutination, for example, is regarded as a measure of adhesion of pathogens to the host which is the first step in colonization. Damage to host cells can be mediated by the haemolysins which are membrane-damaging toxins. Capsule formation is involved with the protection of the organisms from phagocytosis resulting in increased virulence. It has been earlier demonstrated that encapsulated *S. aureus* were more virulent in mice than their unencapsulated counterpart [22]. In addition to this, biofilm formation is an established mechanism of CoNS pathogenicity [23]. Results indicate that these factors may have substantial roles to play in the production of these lesions as the severity of pathological effects were directly proportional to the number and type of virulence factors expressed by the organism [13,20]. Further work should pinpoint the specific virulence determinants responsible for these effects.

The cells of the livers showed congestion, aggregates and multinucleated hepatocytes. All these could be signals of inflammatory process especially the infiltration of portal tract by chronic inflammatory cells. Liver congestion could be as a result of excessive intake or presence of toxins in the blood and this might result in the dysfunction of the liver. Other situation that has been reported to result into the congestion of the liver is in the reduction or stoppage in bile flow. The *S. epidermidis* species might also play some roles in this respect and this is worthy of further investigation.

The kidneys showed from mild to moderate infiltration of the tubule interstitial by chronic inflammatory cells. In some cases, in addition to the mild compression of capillary luminal, there were necroses. Necrosis refers to a spectrum of morphologic changes that follow cell death in living tissues [24]. In renal coagulative necrosis, the cells usually appear pale and ghostlike. There was a haemorrhagic zone in the middle, where the cells were dying or were not quite dead and the normal parenchyma cells were at the far right. These effects were pronounced the most in mice infected with the *S. epidermidis* strain A245A. The three strains of the *S. epidermidis* used in this study apparently did not have the ability to invade some cells in the glomerulli as these appeared not to be affected by their infections.

Coagulative necrosis was observed to be the most common pathological changes in the spleens, intestines and stomachs. These effects could be as a result of infiltration by inflammatory cells. Coagulative necrosis are known to result largely from the progressive degradative action of enzymes on lethally injured cells [24]. Necrosis also occurs in the setting of irreversible exogenous injury. Necrotic cells show increased eosinophilia attributable in part to loss of the normal basophilia imparted by the RNA in the cytoplasm and in part to the increased binding of eosin to denatured structural enzymatic proteins [24].

Coagulative necrosis is the most common type of necrosis with irreversible focal injury, mostly caused by sudden cessation of blood by bacterial or chemical agents [24,25]. It has been earlier reported that *S. aureus* infection in mice cause tissue damage as characterized by inflammatory polymorphonuclear cell infiltration and necrosis of hepatocytes, granulomatous inflammation in the lung and narrowing of the pulmonary alveolar septa [26].

The presence of kidney, liver and peritoneal abscesses and splenomegaly in mice intraperitoneally challenged by three strains of *S. epidermidis* isolated from wound, blood, and urine of inpatients has been demonstrated as early as 1992 [9]. The workers reported splenomegaly as the most frequent macroscopic pathological alteration from strains of this organism. Our results may serve as a confirmation of earlier reports which demonstrated the spleen as a target organ for CoNS [9]. The fact that the *S. epidermidis* strains used in this study were from the human gastrointestinal tracts might indicate the importance of CoNS colonising the intestinal tract in the pathogenesis of the spleen dysfunction.

Another study has reported that the numbers of cases of splenomegaly were directly proportional to LD_50_ of the strains of *S. epidermidis* investigated in that study [27]. This was not the case in this study as lethality could not be demonstrated for most of these organisms at similar dosages and therefore could not be correlated with this condition. However, the dysfunction of the spleen, in addition to other pathological disorders, is likely to be part of what is responsible for the lethal effects seen by sub-chronic infections which caused the death of 52.4% of the mice. Splenomegaly has been reported to be caused by lymphoproliferation and the ability of staphylococcal extracellular slime to stimulate the production of immunologically competent cells [28]. Furthermore, the protein A of *S. aureus* has been reported to cause the depletion of the B cells precursors, splenic marginal zone B cells, resulting in poor generation of specific B cell response [22,29]. Although this role has not been described for *S. epidermidis* the possibility of its occurence remains as this organism is a permanent colonizer of humans and has been judged to cause infection in susceptible individuals [12].

It is to be noted that no pathological lesions were observed in any of the control animals none of which died during the course of the study. This is an indication that the effects observed with the infected animals were all due to the activity of the *S. epidermidis* strains with which they were infected.

## Conclusion

The results of this study suggest that the intestinal colonization with strains of *S. epidermidis* can cause devastating effects on certain organs such as kidney, liver, intestine, stomach and spleen which, depending on their severity, could be fatal. This implies that this organism may play essential roles in the function of the gastrointestinal tract.

## Competing interests

The authors declare that they have no competing interests.

## Authors’ contributions

EOA and AL conceived and designed the study. IOA participated in the design. EOA provided the organisms and prepared them for the animal studies. EOA, IOA and EOO carried out animal studies. OAI was involved in the preparation of isolates for part of animal studies. AEO was involved in the histopathological investigation. EOA coordinated the study and the data management and wrote the manuscript which was revised by AL. All authors approved the final manuscript.

## Pre-publication history

The pre-publication history for this paper can be accessed here:

http://www.biomedcentral.com/1471-230X/14/126/prepub
